# Hybrid Biochar/Ceria Nanomaterials: Synthesis, Characterization and Activity Assessment for the Persulfate-Induced Degradation of Antibiotic Sulfamethoxazole

**DOI:** 10.3390/nano12020194

**Published:** 2022-01-07

**Authors:** Golfo Papatheodorou, Paraskevi Ntzoufra, Evroula Hapeshi, John Vakros, Dionissios Mantzavinos

**Affiliations:** 1 Department of Chemical Engineering, University of Patras, Caratheodory 1, University Campus, GR-26504 Patras, Greece; fofopapatheodorou@gmail.com (G.P.); voula_ntz1994@hotmail.com (P.N.); 2 Department of Life and Health Sciences, School of Sciences and Engineering, University of Nicosia, Nicosia 2417, Cyprus; hapeshis.e@unic.ac.cy

**Keywords:** biochar, emerging contaminants, nanoceria, Fenton-like reaction, SR-AOPs, water treatment

## Abstract

Biochar from spent malt rootlets was employed as the template to synthesize hybrid biochar-ceria materials through a wet impregnation method. The materials were tested for the activation of persulfate (SPS) and subsequent degradation of sulfamethoxazole (SMX), a representative antibiotic, in various matrices. Different calcination temperatures in the range 300–500 °C were employed and the resulting materials were characterized by means of N_2_ adsorption and potentiometric mass titration as well as TGA, XRD, SEM, FTIR, DRS, and Raman spectroscopy. Calcination temperature affects the biochar content and the physicochemical properties of the hybrid materials, which were tested for the degradation of 500 μg L^−1^ SMX with SPS (in the range 200–500 mg L^−1^) in various matrices including ultrapure water (UPW), bottled water, wastewater, and UPW spiked with bicarbonate, chloride, or humic acid. Materials calcined at 300–350 °C, with a surface area of ca. 120 m^2^ g^−1^, were the most active, yielding ca. 65% SMX degradation after 120 min of reaction in UPW; materials calcined at higher temperatures as well as bare biochar were less active. Degradation decreased with increasing matrix complexity due to the interactions amongst the surface, the contaminant, and the oxidant. Experiments in the presence of scavengers (i.e., methanol, t-butanol, and sodium azide) revealed that sulfate and hydroxyl radicals as well as singlet oxygen were the main oxidative species.

## 1. Introduction

Over the last few decades, nanotechnology has gained huge interest due to its extensive application in various fields including, among others, catalysis, electronics, optics, energy, and the environment. The design and controlled synthesis of advanced nanomaterials with unique properties make them highly attractive in these fields. The demand for more active, environmentally friendly, low-cost materials has resulted in tremendous interest in the preparation of nanostructured materials with active surface functional groups and, thus, high surface reactivity. Two very interesting materials with many environmental applications are CeO_2_ [1,2] and biochar [3,4]. The reason for this is their unique characteristics. Specifically, CeO_2_ is a very promising material that can be used as a catalyst or support in several catalytic applications. Combined with Cu is the state-of-the-art catalyst for the preferential oxidation of CO [5] since it presents a high amount of oxygen vacancies, a controllable ratio of Ce^3+^/Ce^4+^, high oxygen storage capacity, and moderate surface area [2,6,7].

Nano CeO_2_ may exhibit improved properties and catalytic functions, which are significantly affected by the preparation conditions [8]. It has been reported in the literature and demonstrated by computational studies that the morphology of the nano CeO_2_ affects the catalytic activity. Generally, the (110) and (100) surfaces are catalytically more active than (111) [9,10]. Specifically, the higher activity for the CO oxidation can be obtained with nanorods of CeO_2_, which are exposed to the (110) and (100) planes [11]. The hydrothermal method can be applied for the preparation of different forms of nano CeO_2_ [2].

The main disadvantage of CeO_2_ is that pure ceria exhibits low activity since the active surface oxygen species are rather limited; moreover, CeO_2_ is an insulator at room temperature, thus electron transfer is limited [12]. This can be dramatically changed if pure CeO_2_ is doped with transition metal ions. Then, the deposition of metal cations (M) on CeO_2_ can alter the electronic and geometric configuration and thus the properties of the mixed material. This approach is well-studied and researchers have concluded that new sites with high activity can be formed, interactions between Ce and M can alter the electrons configuration, and new oxygen vacancies can be formed during sub-surface and bulk incorporation of metal ions into CeO_2_ [13,14,15].

In a recent review, the synthesis and characterization of CeO_2_ nanoparticles of different morphology were discussed [16]. Although many methods have been applied for the synthesis of CeO_2_ nanoparticles, the combined use of biochar (BC) as a template and Ce precursors for the preparation of CeO_2_ has not been investigated. On the other hand, CeO_2_ deposition on biochar to produce hybrid materials with enhanced adsorption capacity [17], interesting electrocatalytic properties [18], or superior degradation ability for textile dyes in sonocatalytic oxidation [19] has been reported; in these cases, CeO_2_ nanoparticles were prepared with the hydrothermal method and then mixed with BC as a suspension in acetone. Moreover, novel carbonaceous materials (i.e., CeO_2_-encapsulated nitrogen-doped biochar) have significant activity for oxygen reduction [20]. The biomass precursor of this value-added biochar material was biomimetically prepared via a hydroponic operation in the Ce-enriched solution. The enhanced activity was partially due to high oxygen vacancies of the hybrid material.

Biochar is the solid material prepared from the pyrolysis of biomass under a limited or no oxygen atmosphere, during which part of the organic phase decomposes to gases. Interestingly, the properties of the biochars are different compared to raw biomass. These new unique properties are desirable for many applications where biochars are employed as absorbers [21], support in catalytic processes [22,23,24], transesterification catalysts for the production of biodiesel, [25], supercapacitors [26], and persulfate activators for the oxidation of organic contaminants in water [27,28,29,30]. Depending on the raw biomass and pyrolysis conditions, biochars can exhibit high surface area, hierarchical pore structure, plenty of surface groups, stability, and in some cases, a high amount of mineral deposits. Furthermore, its surface can interact with metal ions or even nanoparticles and stabilize them.

Pharmaceutically active compounds such as antibiotics, antihypertensive and non-steroidal anti-inflammatory drugs as well as their metabolites are classified as emerging micro-contaminants and are detected in wastewater, surface water, and groundwater at concentrations ranging from ng L^−1^ to mg L^−1^. Antibiotics are widely used against bacterial infections or to prevent infections; several studies have reported antibiotic occurrence in wastewater treatment plant effluents at concentrations from 0.1 to 2.5 μg L^−1^ [31]. Although this range of concentration is small, it can be harmful to human and animal health and augment the antimicrobial resistance [32].

In this work, biochar from malt spent rootlets was prepared at 850 °C. This biochar has moderate surface area and a high amount of minerals [33]. Treatment with H_2_SO_4_ can remove the minerals and significantly increase the specific surface area [34]. For this reason, the biochar was treated with H_2_SO_4_ and then used as a template for the preparation of hybrid material BC-CeO_2_ with different BC to CeO_2_ ratio. The produced materials were characterized with various physicochemical methods and used for the degradation of sulfamethoxazole, a representative antibiotic drug, in various water matrices via oxidation with persulfates. To the best of our knowledge, this is the first report on (i) the use of biochar as a template and its influence on the physicochemical properties of CeO_2_, and (ii) the application of the as-prepared hybrid materials to promote the sulfate radical-induced advanced oxidation of antibiotic SMX in environmentally relevant matrices. From this perspective, the innovation of this work embodies two different but related disciplines, namely (i) material synthesis, where a novel catalytic material capable of activating persulfate is described, and (ii) environmental remediation, focusing on the treatment of micro-contaminants of emerging concern.

## 2. Materials and Methods

### 2.1. Materials

The precursor salt Ce(NO_3_)_3_·6H_2_O (analytical grade, CAS number: 10277-43-7) and sulfamethoxazole (SMX, C_10_H_11_N_3_O_3_S, 99+%, CAS number: 723-46-6) were purchased from Sigma-Aldrich (St. Louis, MO, USA). Sodium persulfate (SPS, Na_2_S_2_O_8_ 99%, CAS number: 7775-27-1) was purchased from Scharlau (Barcelona, Spain). Most of the experiments were carried out in ultrapure water (UPW: pH = 6.5). Other matrices included (i) commercially available bottled water (BW: pH = 7.7, conductivity 355 μS cm^−1^, containing (in mg L^−1^): 237 bicarbonate; 3.7 chloride; 7.8 sulfate; 1.1 nitrate; 75.5 calcium; 5.1 magnesium; 2.1 sodium; and 0.65 potassium ions); (ii) secondary treated wastewater (WW) taken from the University of Patras campus treatment plant (pH = 8, conductivity = 1.682 mS cm^−1^, total organic C = 2.46 mg L^−1^, chemical oxygen demand = 48.53 mg L^−1^, total suspended solids = 22 mg L^−1^, [Cl^−^] = 262.41 mg L^−1^, [PO_4_^3^^−^] = 14.98 mg L^−1^, [HCO_3_^−^] = 278 mg L^−1^, [Br^−^] = 165.64 mg L^−1^, [Ca^2+^ ] = 112 mg L^−1^); and (iii) UPW spiked with various water constituents such as humic acid (HA: CAS number: 1415-93-6), bicarbonate (CAS number: 144-55-8), chloride (CAS number: 7647-14-5), sodium azide (NaN_3_: CAS number: 26628-22-8), t-butanol (CAS number: 75-65-0), and methanol (CAS number: 67-56-1); all these were purchased from Sigma-Aldrich (St. Louis, MO, USA).

### 2.2. Sample Preparation

The biochar used in this study was prepared from spent malt rootlets under pyrolysis at 850 °C with limited O_2_ atmosphere. The prepared biochar was treated with 1 M H_2_SO_4_ under reflux for 30 min. The treatment was conducted in order to increase the surface area of the sample and remove the deposits of minerals present in the raw biomass. After treatment, the BC was filtered, washed with 1 L of triply distilled water, and dried for 2 h at 120 °C. More details on the preparation of the sample and its properties can be found in [34].

To deposit the Ce precursor on the biochar surface, about 3 g of treated biochar was immersed in 150 mL of solution containing 11.1 g of Ce(NO_3_)_3_ 6H_2_O. The suspension was placed in a round bottom bottle in a rotary evaporator system. Then, the suspension was left to equilibrate for 30 min under atmospheric pressure at 70 °C. After that, vacuum was applied and the water was evaporated. The mixed solid was dried at 120 °C for 1 h and then calcined at different temperatures for 2 h. The samples were denoted as BC/Ce-X, where X is the calcination temperature. An additional sample (BC/Ce-300-5 h) was calcined at 300 °C for 5 h.

### 2.3. Physicochemical Characterization

The prepared samples were characterized with various physicochemical methods. Briefly, specific surface area (SSA) and pore size distribution was performed with N_2_ adsorption isotherms at liquid N_2_ temperature in a Tristar 3000 porosimeter (Micromeritics). X-ray diffraction peaks were recorded with a Bruker D8 Advance diffractometer (Billerica, MA, USA) equipped with a nickel-filtered CuKa (1.5418 Å) radiation source. The biochar morphology was examined by scanning electron microscopy (SEM JEOL JSM6300) equipped with EDS. Fourier transform infrared analysis was performed in a Perkin Elmer Spectrum RX FTIR system (Waltham, MA, USA). The samples were diluted in KBr (1% *w*/*w* sample) and pressed in pellet form with 8 atm pressure. The point of zero charge was determined using the potentiometric mass titration method [35]. A suspension of 0.1 g in 75 mL of NaNO_3_ 0.03 M was titrated with 0.1 M HNO_3_ from pH 11 to 2 and the titration curve was compared with the corresponding curve of the solution. The section point of the two curves is the point of zero charge of the solid sample. The thermogravimetric analysis of the samples was performed in a TGA Perkin Elmer system (Waltham, MA, USA) under an air atmosphere with a flow of 20 mL min^−1^. The heating rate was 10 °C min^−1^ in the temperature range of 80–700 °C. Diffuse reflectance spectroscopy (DRS) was performed using a UV–Vis spectrophotometer (Varian Cary 3) equipped with an integration sphere. The spectra of the solid samples were recorded in the range of 200–800 nm using PTFE or commercial CeO_2_ as references. The powder samples were mounted in a quartz cell, which provided a sample thickness >3 mm to guarantee the “infinite” sample thickness. Raman spectra were taken on a Micro Raman Spectroscopy system (Jobin–Yvon Horiba LabRam–HR) with a 514 nm line of an Ar ion laser at room temperature. A 50× microscope objective lens was used to focus the laser beam and collection of the scattered light. Typical spectrum acquisition time was 5 s.

### 2.4. Catalytic Activity

A stock solution of SMX (50 mg L^−1^) in UPW was prepared and used for all the catalytic tests. In a typical run, 120 mL of an aqueous solution containing 500 μg L^−1^ SMX and 90 mg L^−1^ BC were loaded into a beaker under stirring at ambient temperature. After 20 min of equilibration, SPS was added. Samples of 1.2 mL were periodically drawn from the reactor, an excess of methanol was added (5 mol L^−1^) to quench the reaction and the samples were filtered and analyzed using high-performance liquid chromatography (HPLC) (Waters Alliance 2695, Waters 2996 Milford, PA, USA). More details about the catalytic tests and analysis can be found in [36].

## 3. Results and Discussion

### 3.1. Samples Characterization

The biochar from malt spent rootlets had a moderate specific surface area, SSA, of 100 m^2^ g^−1^ a point of zero charge, pzc, equal to 8.2 and 32% minerals. Following treatment with H_2_SO_4_, the concentration of minerals diminished since they were soluble in acidic solution, while SSA increased considerably to 428 m^2^ g^−1^. The microporosity was also high (190 m^2^ g^−1^) and the pzc shifted to more acidic values. These properties make the treated biochar an ideal candidate for the preparation of hybrid ceria–biochar materials. The low value of pzc ensures a positively charged surface, where cations can be deposited. The high SSA favors the adsorption of considerable quantities of cations, while the micropores can prevent the formation of bulk precipitates during drying. The deposition of Ce ions can easily be performed with wet impregnation, during which Ce(III) ions interact with the biochar surface through electrostatic adsorption because of the low value of pzc. The high value of SSA and microporosity allows for the deposition of well-dispersed Ce particles. Finally, the calcination process can provide the possibility of controlling the biochar content and, in parallel, to convert the Ce precursor form to CeO_2_ nanoparticles. With the regulation of calcination temperature, one can prepare hybrid materials BC–CeO_2_ with different biochar contents.

The prepared samples, alongside their properties, are presented in Table 1.

Interestingly, increasing the calcination time from 2 to 5 h at 300 °C did not practically alter the properties of the prepared samples. Although longer calcination times were not tested at higher temperatures (where the BC content is lower), one could possibly expect to see detrimental effects on physicochemical properties such as SSA, Eg, and oxygen vacancies due to extensive sintering.

**Figure 1:**
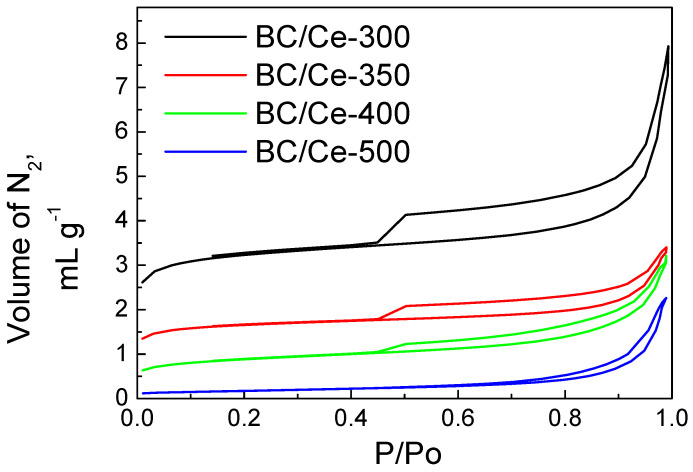
The N_2_ adsorption–desorption isotherms for the prepared samples.

The adsorption/desorption isotherms for the hybrid samples are presented in Figure 1. The SSA was lower at higher temperatures of calcination. The samples calcined at 300–400 °C exhibited type IV with an H4 hysteresis loop, while for the BC/Ce-500 sample, the hysteresis loop was between H3 and H4. H4 loops are often found in micro-meso porous carbon materials [37]. The BC/Ce-500 sample also exhibited limited N_2_ adsorption at low P/Po values, suggesting low microporosity, in contrast with the other three samples. SSA values were higher than the commercial CeO_2_ with a SSA of 4 m^2^ g^−1^.

**Figure 2:**
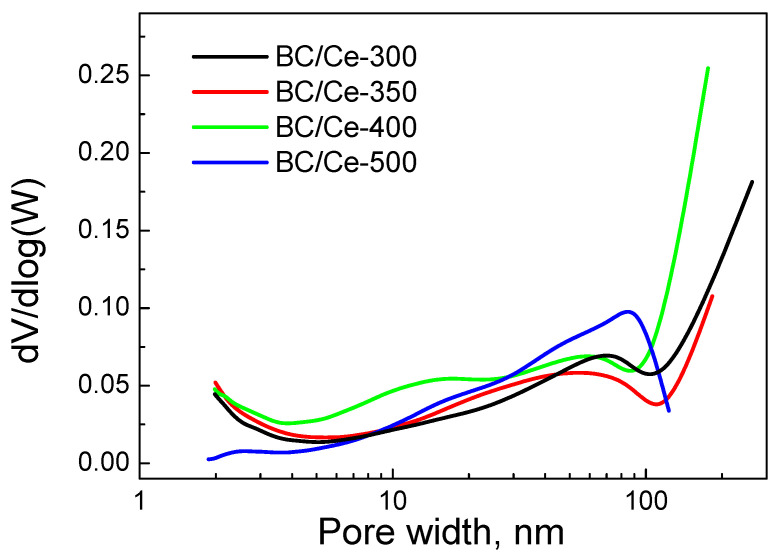
Pore size distribution for the studied samples.

This was confirmed by the pore size distribution shown in Figure 2. The BC/Ce-500 had a limited amount of micropores (if any), in contrast with the other samples calcined at lower temperatures. For the BC/Ce-500 sample, there was a peak centered at about 85 nm, while the main peak was at 60 nm for the other samples; an additional peak was centered at 13 nm for BC/Ce-400. This increment in pore diameter was due to higher calcination temperature and the collapse of microporosity.

**Figure 3:**
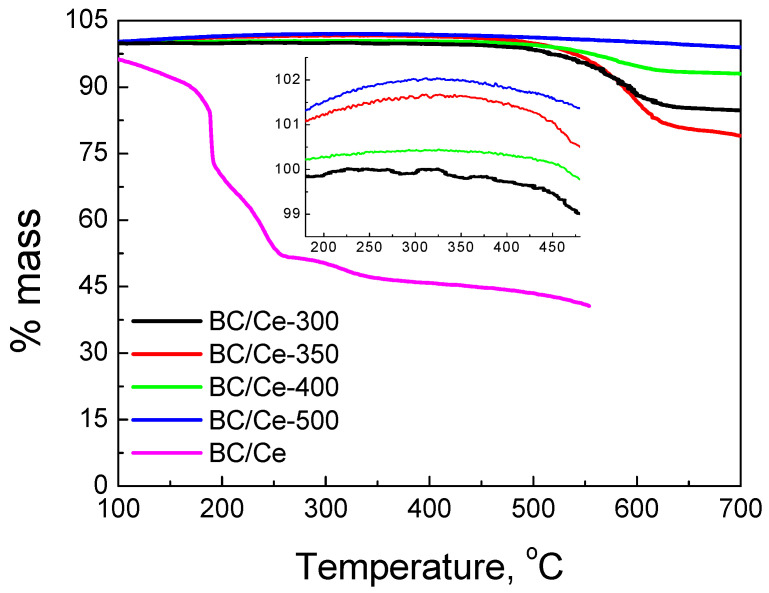
TGA curves under an air atmosphere for the studied samples as well as the uncalcined one.

The TGA curves for the prepared samples as well as the mixed material before calcination (BC/Ce) are presented in Figure 3. The % mass left after TGA was due to the CeO_2_ content in each hybrid material and the difference from the starting mass is characteristic of the biochar content.

As can be seen, the amount of mass left after TGA was significant and depends on the calcination temperature (see also Table 1). This means that the amount of biochar left after calcination was limited and the samples mostly consisted of CeO_2_. The biochar content was 22% for the BC/Ce-350 sample and 16% for the BC/Ce-300 sample, while the other two samples calcined at higher temperatures had an even lower content. Although the BC/Ce-300 sample should be expected to have a higher BC content than the BC/Ce-350 sample, this discrepancy may be attributed to the low difference in the respective calcination temperatures.

For the starting material before calcination, there was first sharp mass decrease at 185 °C, which was followed by a second step at 190 °C. This step was completed at about 260 °C, while the mass was quite stable at higher temperatures. Therefore, a temperature up to 300 °C seems to be sufficient to transform the precursors to CeO_2_ nanoparticles. The mass left at temperatures higher than 550 °C was about 42% of the starting mass, very close to the value of the nominal CeO_2_ content (41%), which implies that the starting material is transformed to CeO_2_ at temperatures up to 500 °C.

**Figure 4:**
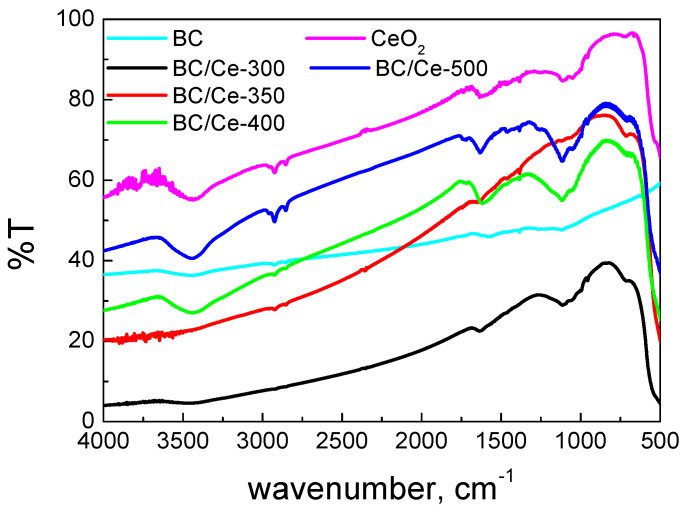
FTIR spectra for the studied samples as well as the starting biochar and a commercial CeO_2_ sample.

The FTIR spectra of the prepared samples are presented in Figure 4, together with the spectra of BC and commercial CeO_2_. The FTIR peaks were more intense in the prepared materials in contrast with the commercial CeO_2_. This may be due to the different SSA values, which were higher in the prepared samples, suggesting the existence of more surface groups. On the other hand, the BC/Ce-300 and BC/Ce-350 samples had peaks with low intensity, suggesting that CeO_2_ was less bulk in these samples.

**Figure 5:**
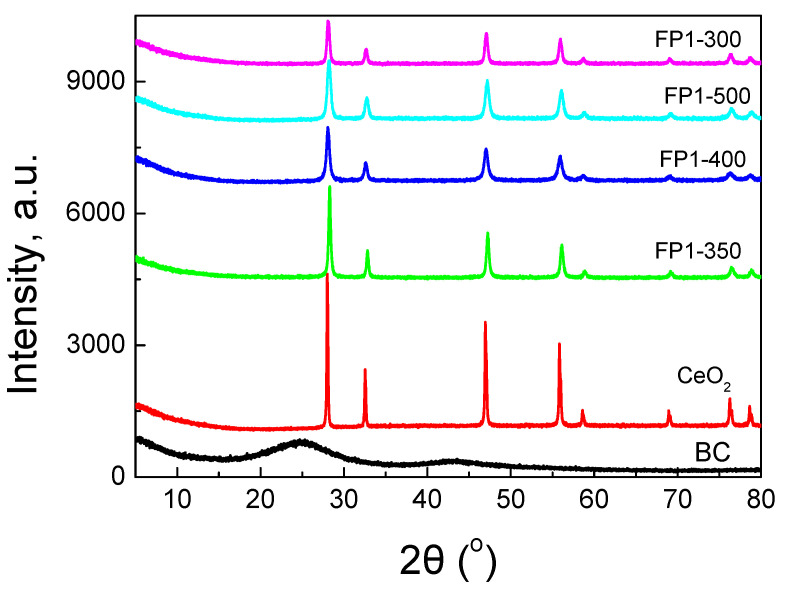
XRD patterns for the studied samples as well as the starting biochar and a commercial CeO_2_ sample.

The XRD patterns of the prepared samples, alongside the BC and commercial CeO_2_, are presented in Figure 5; the main peaks can be evidently attributed to CeO_2_. The average diameter of the CeO_2_ particles calculated from the Scherrer equation varied from 29.7 to 16 nm, while for the commercial CeO_2_, it was 46.2 nm.

**Figure 6:**
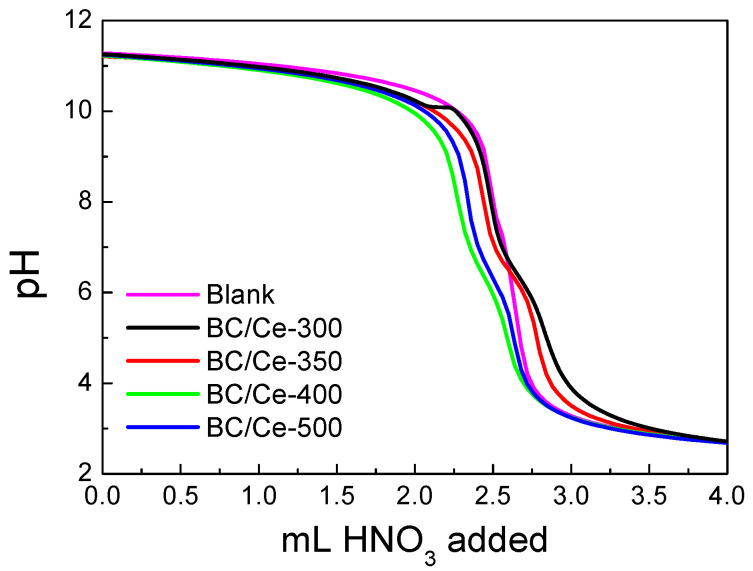
Potentiometric mass titration curves for the studied samples as well as the corresponding solution titration curve.

The acid–base behavior of the samples is presented in Figure 6. The potentiometric titration curves revealed that the two materials prepared at the lower calcination temperatures had a pzc near 7 (6.8 and 6.5 for the BC/Ce-300 and BC/Ce-350, respectively), while the two other samples had a pzc value equal to 3, pointing out the acidity of these samples. Generally, low pzc values have been reported for CeO_2_ when the precursor is Ce(III) salt, as in our case [38].

**Figure 7:**
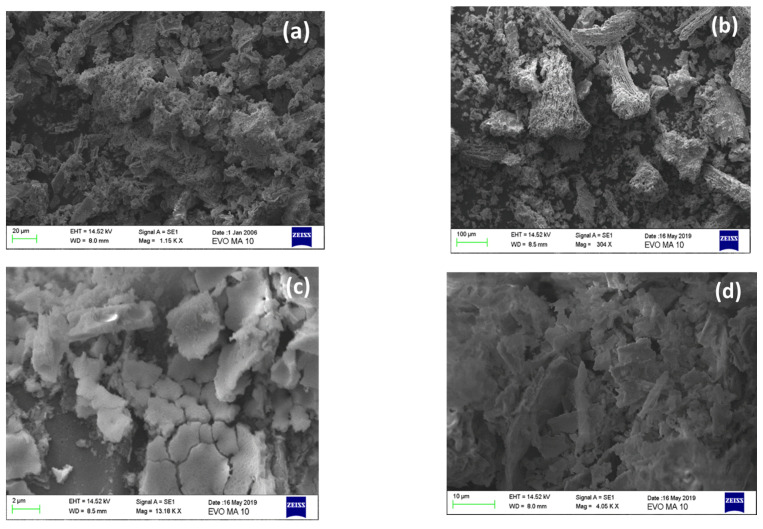
SEM images for (**a**) BC/Ce-300, (**b**) BC/Ce-350, (**c**) BC/Ce-400, and (**d**) BC/Ce-500.

SEM images of the prepared samples are presented in Figure 7, where the progressive removal of BC and the transformation to a more solid CeO_2_ phase could be observed. For the BC/Ce-350 sample (Figure 7b), BC could clearly be seen, while CeO_2_ formed around the carbon particles. This was less pronounced in the case of BC/Ce-300 (Figure 7a), although the calcination temperature was lower. There probably exists a minimum calcination temperature needed for the formation of CeO_2_ particles and the simultaneous burning of biochar.

On the other hand, higher calcination temperatures lead to greater removal of the carbon phase, and thus CeO_2_ is better formed. Carbon removal in the form of volatile compounds results in CeO_2_ cracking and this facilitates the formation of the surface area of the sample; this can be seen in Figure 7c for the BC/Ce-400 sample. Even higher calcination temperatures may result in sintering of the CeO_2_ particles and, eventually, greater degree of agglomeration (Figure 7d). These findings are in accordance with the SSA values (Table 1) and the XRD results.

**Figure 8:**
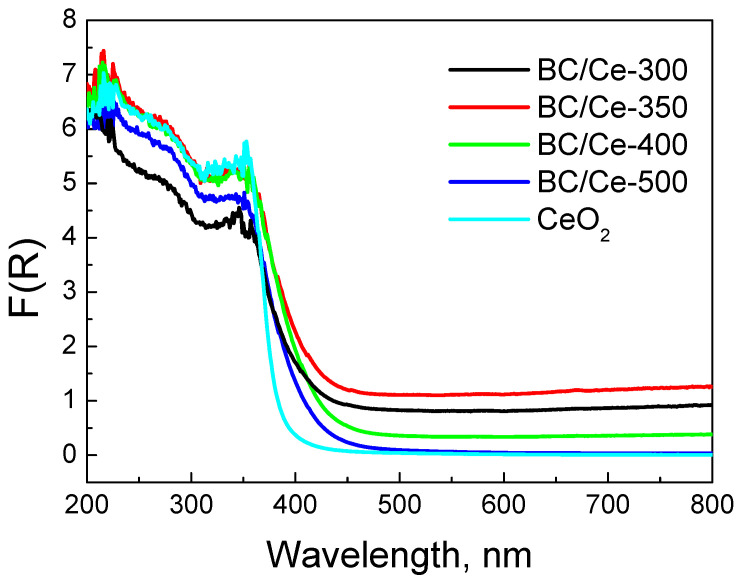
DR spectra for the studied samples as well as a commercial CeO_2_ sample. The spectra were collected with PTFE disks as the reference.

The DR spectra of the prepared samples are similar to that of CeO_2_, especially at wavelengths lower than 400 nm (Figure 8). In the UV and Vis near UV regions, the peaks are due to charge transfer between the Ce(IV) and O^2−^ species. The DR spectrum of CeO_2_ showed three maxima at about 220, 270, and 330 nm. The peak at 220 nm was assigned to *f-d* transition of Ce(II), the band at 270 nm to surface sites, and the band at 330 nm to bulk sites [39,40,41,42]. The exact location of the peaks is highly influenced by the size of CeO_2_ crystallites. At wavelengths higher than 400 nm, CeO_2_ exhibits negligible absorbance and this is also the case for BC/Ce-500 (i.e., the sample with the minimum BC content). The other three samples exhibited a constant absorbance over the whole range of the Vis spectrum, which was due to the black color of the biochar. The absorbance intensity was well correlated to the BC content, with the BC/Ce-350 sample showing the higher intensity. For the prepared samples, the point where a sharp increase in absorbance occurred shifted at higher wavelengths. This implies a change in the distribution of electrons and can be confirmed from the energy gap values for each material. These values were between 3.08–3.12 eV for all the prepared samples and 3.32 eV for commercial CeO_2_ (Table 1). The observed red shift in the absorbance of the samples is correlated to the smaller size of CeO_2_ nanoparticles, in contrast with the commercial CeO_2_.

**Figure 9:**
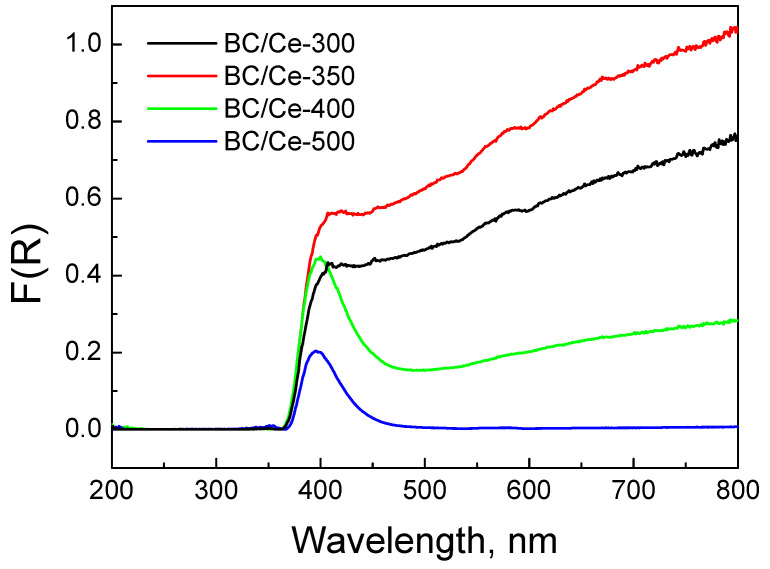
DR spectra for the studied samples. The spectra were collected with commercial CeO_2_ as reference.

Changes in electrons distribution are more clearly demonstrated in Figure 9, where commercial CeO_2_ was employed as the reference. Indeed, the peak centered at about 400 nm revealed that interactions were more pronounced for the samples with increased BC content.

**Figure 10:**
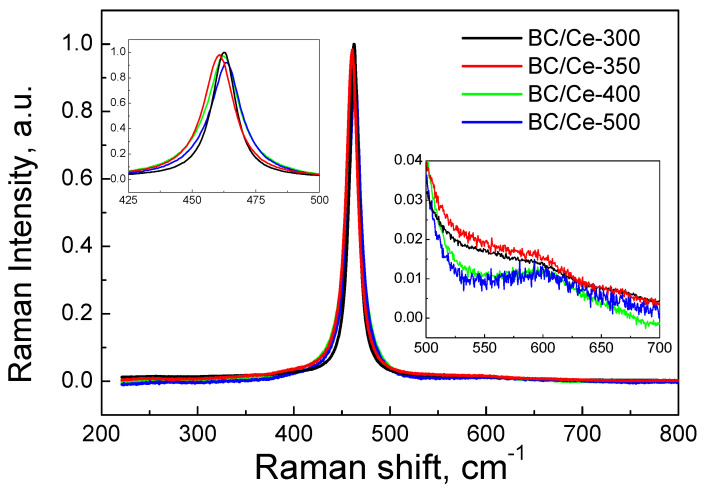
Normalized Raman spectra for the studied samples.

Figure 10 shows the normalized Raman spectra for the prepared samples. There was an intense peak centered between 460 and 464 cm^−1^, which corresponded to the F2g Raman vibrational mode of cubic fluorite lattice of CeO_2_ [39,43,44]. The broadness and asymmetry of the peak imply the existence of nanosized CeO_2_ particles. The particle size as well as possible changes in composition associated with varying BC content influence the exact position and the broadening of the peak with a shift toward lower frequencies. There was another broad peak at about 600 cm^−1^, which can be attributed to oxygen vacancies of CeO_2_ [44,45]. It is interesting to note that this peak was broader and more intense for the samples with higher BC content.

### 3.2. Assessment of Catalytic Activity for the Degradation of SMX

**Figure 11:**
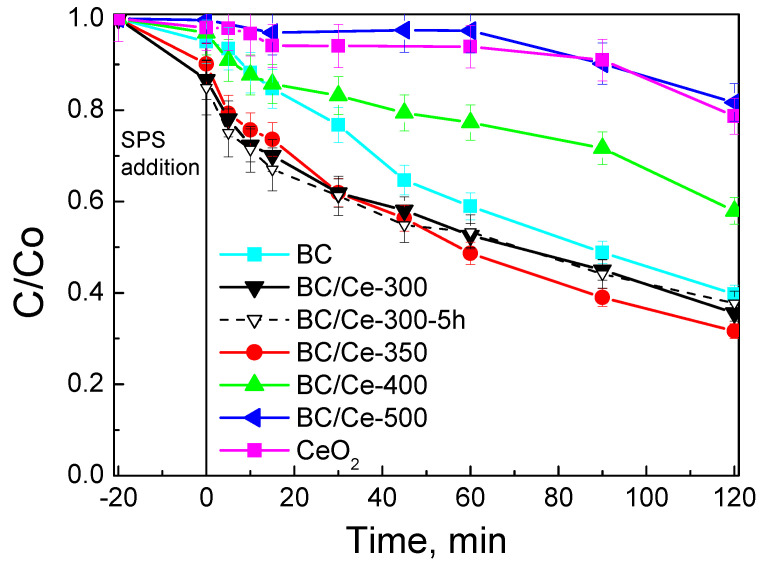
Screening of various materials (90 mg L^−1^) for the removal of 500 μg L^−1^ SMX with 200 mg L^−1^ SPS in UPW and ambient pH.

Figure 11 shows the relative catalytic activity of the various prepared samples as well as bare biochar and ceria. The time-scale corresponded to the equilibration period where only adsorption occurred, followed by the oxidative degradation period. It must be clarified here that a common equilibration period of 20 min was employed in this work irrespective of the specific experimental conditions (i.e., type of BC-Ce material, SPS concentration, water matrix, etc.), which implies that the level of SMX adsorption might have not been completed during this period; however, the main scope of this work was to study the oxidative rather than the adsorptive removal of SMX. Commercial ceria is not capable of activating SPS and neither is BC/Ce-500, which contained only 2% BC and whose SSA was 14 m^2^ g^−1^ (Table 1); however, activity seems to increase with increasing BC content and SSA (i.e., 7%–69 m^2^ g^−1^, 16%–119 m^2^ g^−1^, and 22%–126 m^2^ g^−1^ for BC/Ce-400, BC/Ce-300, and BC/Ce-350, respectively. Bare BC with a SSA of 428 m^2^ g^−1^ exhibited good activity relative to that of BC/Ce-300 and BC/Ce-350 samples, although the hybrid samples have lower SSA. An additional run was performed with BC that had not been acid-treated (data not shown for brevity); its performance was comparable to that of the acid-treated sample. Considering that SMX degradation can be modeled by a pseudo-first order kinetic expression, data in Figure 11 can be employed to compute the apparent rate constants, k. The relative activity decreases in the order: BC/Ce-350 (8.9 × 10^−3^) ≈ BC/Ce-300 (8.2 × 10^−3^) > BC (7.4 × 10^−3^) > BC/Ce-400 (4.6 × 10^−3^) > CeO_2_ (1.4 × 10^−3^) ≈ BC/Ce-500 (1.3 × 10^−3^), with numbers in brackets corresponding to k values expressed in min^−1^. The dashed line (open symbols) shown in Figure 11 corresponds to a run with the BC/Ce-300 material that had been calcined for 5 h; interestingly, its catalytic activity was similar to that of the material calcined for 2 h (k = 7.8 × 10^−3^), implying that the calcination temperature rather than time is the crucial factor. This finding is also consistent with the similar properties of the two samples, as shown in Table 1.

Based on the results shown in Figure 11, subsequent activity tests were performed with the BC/Ce-350 material.

**Figure 12:**
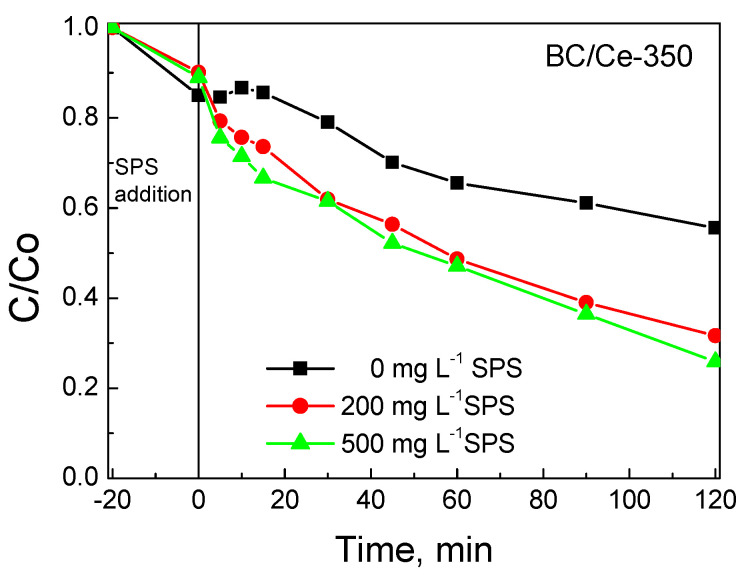
Effect of SPS concentration on 500 μg L^−1^ SMX removal with 90 mg L^−1^ BC/Ce-350 in UPW and ambient pH.

The influence of SPS concentration on SMX degradation is presented in Figure 12. In the absence of SPS, SMX removal can occur to a considerable extent due to adsorption only (i.e., 45% at the end of the experiment). The addition of SPS in the range 200–500 mg L^−1^ promotes SMX removal due to the reactions occurring between the generated radicals and SMX. Interestingly, the rate was not affected by the level of oxidant concentration used with the apparent rate constant being 9.1 ± 0.1 10^−3^ min^−1^; this value was about 2.5 times greater than that of pure adsorption.

It must be noted here that the minimum effective level of persulfates employed in environmental applications depends on several factors including the type of activation (homogeneous or heterogeneous), the recalcitrance of the contaminant under consideration, the quality of the aquatic phase and, in the case of chemical activators such as transition metals, carbocatalysts, etc., their concentration. On the other hand, there always exists an upper concentration threshold, above which persulfate may act as a self-scavenger and/or introduce secondary water pollution due to the release of sulfate salts in the environment [46]. From a managerial point of view, solid persulfate is more advantageous than liquid hydrogen peroxide employed in traditional Fenton chemistry since it is more stable, easier to handle, store, and transport, and has a lower market price.

**Figure 13:**
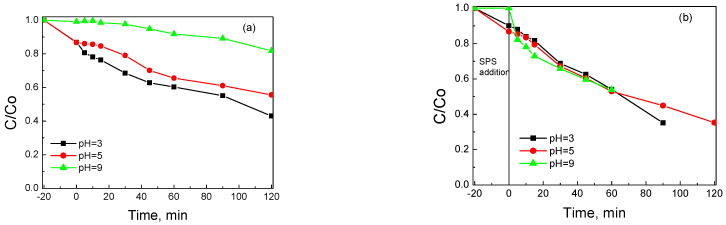
Influence of solution pH on (**a**) adsorption without SPS and (**b**) degradation with 200 mg L^−1^ SPS of 500 μg L^−1^ SMX on 90 mg L^−1^ BC/Ce-350.

Since the pzc value of BC/Ce-350 was 6.5 (Table 1), its surface was slightly positive at an ambient pH of 5, which favors the attraction of the negatively charged S_2_O_8_^2−^ anions. SMX, on the other hand, was neutral at pKa_1_ = 1.77 < pH < pka_2_ = 5.65 [29,47,48,49]. SMX was positively charged at pH < 1.4 (protonation of –NH_2_ group) and negatively charged at pH > 5.65 (deprotonation of –NH). To assess the effect of the initial solution pH on SMX removal, experiments were performed by adjusting the ambient pH = 5 to more acidic (pH = 3) or basic conditions (pH = 9), and the results are shown in Figure 13a for adsorption in the absence of an oxidant and 13b for oxidative degradation.

Lower pH values seem to favor SMX adsorption, which is limited at alkaline conditions where both the surface and SMX are negatively charged; the respective k values are 1.4 × 10^–3^, 3.9 × 10^–3^, and 5.4 × 10^–3^ min^–1^ at pH 9, 5, and 3, respectively. Unlike adsorption, the oxidative degradation of SMX does not seem to be affected by the initial solution pH (Figure 13b), with the k value being about 8.9 × 10^–3^ min^–1^ for all three experiments. This is probably due to the fact that the solution pH is not buffered and there is a fast pH decrease from the initial value of 9 or 5 to 6 or 3, respectively, upon the addition of SPS, whose activation initiates oxidation reactions as well as generates HSO_4_^–^, a moderate acid. For the run performed at pH = 3, this value did not change throughout the course of the reaction. In this respect, unbuffered systems are beneficial since the spontaneous pH shift to lower values favors SMX removal. The influence of pH on the adsorption and degradation of SMX was similar to that in a previous work [29], where biochar from spent coffee grounds was employed as a SPS activator.

**Figure 14:**
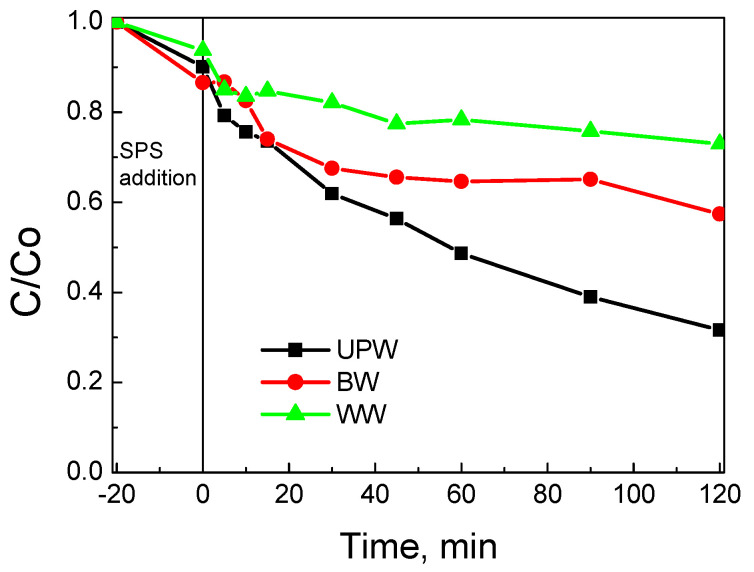
Effect of water matrix on 500 μg L^−1^ SMX removal with 90 mg L^−1^ BC/Ce-350 and 200 mg L^−1^ SPS at ambient pH.

The effect of water matrix on SMX removal is depicted in Figure 14. Reactivity decreased with increasing matrix complexity (i.e., UPW (8.9 × 10^−3^) > BW (3.2 × 10^−3^) > WW (1.6 × 10^−3^)), with numbers in brackets showing k values in min^−1^. The role of the water matrix is associated with the various inorganic and organic, non-target species that are inherently present and may compete with SMX for the active catalytic sites and/or the oxidant.

**Figure 15:**
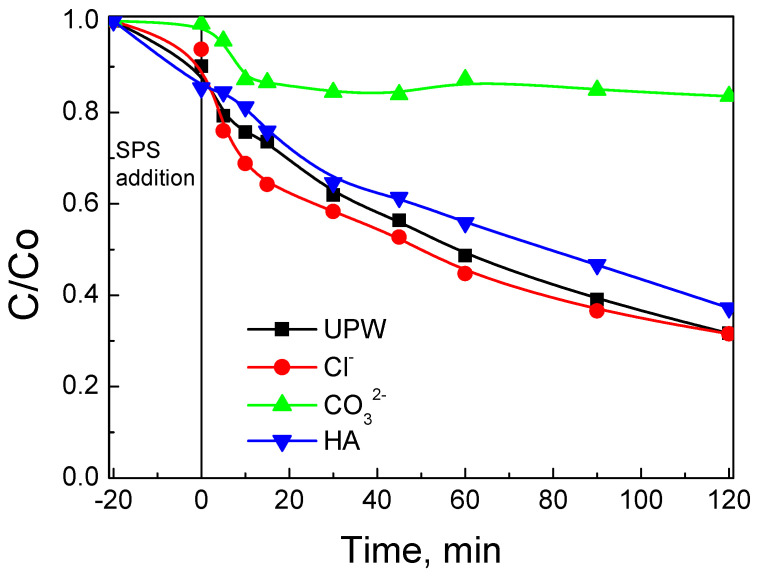
Effect of inorganic ions and humic acid on 500 μg L^−1^ SMX removal with 90 mg L^−1^ BC/Ce-350 and 200 mg L^−1^ SPS in UPW at ambient pH.

To shed light on such interplays, experiments were conducted in UPW spiked with various non-target species and the results are shown in Figure 15. The addition of 250 mg L^−1^ bicarbonate (this concentration is typical for BW) seriously impedes SMX removal, leading to a k value of 10^−3^ min^−1^ (i.e., nine times lower than in UPW). A possible explanation involves the detrimental role of carbonate ions that may (i) occupy catalytic sites, thus reducing SMX adsorption (as can be seen in Figure 15), and (ii) scavenge hydroxyl and sulfate radicals, while forming the less active carbonate radicals, in other words,

(1) $${\mathrm{CO}}_{3}^{2-}+{\mathrm{HO}}^{•}⇆{\mathrm{CO}}_{3}^{•-}+{\mathrm{HO}}^{-}$$

(2) $${\mathrm{HCO}}_{3}^{2-}+{\mathrm{HO}}^{•}⇆{\mathrm{CO}}_{3}^{•-}+H_{2}O$$

(3) $${\mathrm{SO}}_{4}^{•-}+{\mathrm{HCO}}_{3}^{-}⇆{\mathrm{SO}}_{4}^{2-}+{\mathrm{HCO}}_{3}$$

(4) $${\mathrm{SO}}_{4}^{•-}+{\mathrm{CO}}_{3}^{2-}⇆{\mathrm{SO}}_{4}^{2-}+{\mathrm{CO}}_{3}^{•-}$$

The addition of 250 mg L^−1^ chloride has practically no effect on degradation although hydroxyl and sulfate radicals may now react with chloride to form various Cl-containing radicals, in other words,

(5) $${\mathrm{SO}}_{4}^{•-}+{\mathrm{Cl}}^{-}⇆{\mathrm{SO}}_{4}^{2-}+{\mathrm{Cl}}^{•}$$

(6) $${\mathrm{Cl}}^{•}+{\mathrm{Cl}}^{-}⇆{\mathrm{Cl}}_{2}^{•-}$$

(7) $${HO}^{•}+{\mathrm{Cl}}^{-}⇆{\mathrm{ClHO}}^{•-}$$

(8) $$4{\mathrm{Cl}}_{2}^{•-}⇆2{\mathrm{Cl}}^{-}+{\mathrm{Cl}}_{2}$$

(9) $${\mathrm{Cl}}^{•}+H_{2}O⇆{\mathrm{ClHO}}^{•-}+H^{+}$$

(10) $${\mathrm{Cl}}_{2}^{•-}+H_{2}O\to {\mathrm{ClHO}}^{•-}+H^{+}+{\mathrm{Cl}}^{-}$$

In a final test, UPW was added to 10 mg L^−1^ humic acid (HA), an analogue of the organic matter typically found in natural waters (the chosen concentration corresponded to the organic carbon content of WW). The effect of HA was mildly negative, leading to a k value of 7 × 10^−3^ min^−1^; HA, a recalcitrant molecule against chemical oxidation, is likely to competitively consume oxidants as well as occupy catalytic sites, both of which are detrimental to SMX degradation.

**Figure 16:**
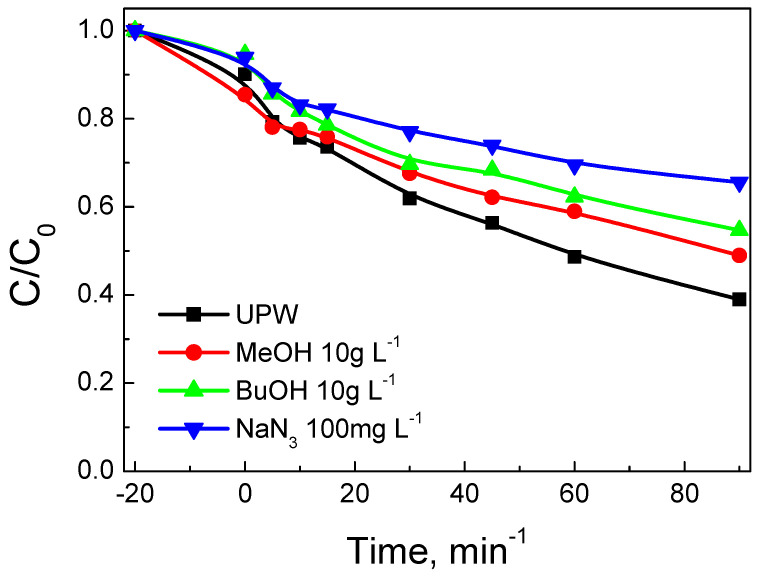
Effect of methanol, t-butanol, and sodium azide on 500 μg L^−1^ SMX degradation with 90 mg L^−1^ BC/Ce-350 and 200 mg L^−1^ SPS in UPW at ambient pH.

Finally, the role of different scavengers was investigated (Figure 16). The addition of methanol (that reacts with both hydroxyl and sulfate radicals) or t-butanol (that preferentially reacts with hydroxyl radicals) at 10 g L^−1^ retarded SMX degradation with the apparent rate constant being 5.7 ± 0.1 × 10^–3^ min^–1^ (i.e., a 35% decrease compared to the run in UPW). This implies that other species are also involved in the degradation mechanism. An additional experiment was performed adding 100 mg L^−1^ NaN_3_, a well-known scavenger for singlet oxygen [50]; in this case, the k value decreased to 3.7 × 10^–3^ min^–1^, thus pointing out the crucial role of active oxygen species in the degradation process. As a matter of fact, the surface oxygen atoms of CeO_2_ may participate in the reaction, which is consistent with the high quantity of surface oxygen vacancies in the BC/Ce-350 sample, as has been demonstrated by TGA and Raman spectroscopy.

**Figure 17:**
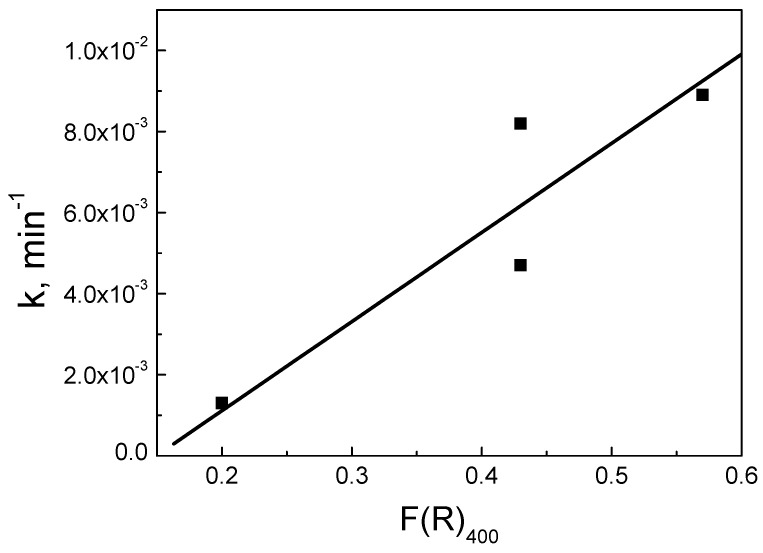
Correlation of k values with the absorbance of the BC/Ce samples at 400 nm. Experimental conditions as shown in Figure 11.

Moreover, the interactions with biochar are important, as can be seen from the DR spectra (Figure 9). Such interactions change the electron distribution in CeO_2_ and regulate the surface reactivity. Figure 17 shows a correlation between the F(R) values of the prepared samples at 400 nm and the k values computed from the data of Figure 11. There appears to be a linear dependence, which implies that interactions are related to the occupied oxygen vacancies in CeO_2_ by the –OH groups of the CeO_2_ surface and/or the active oxygen species formed during SPS activation. The observed correlation suggests that the main active surface sites are these species, in accordance with the detrimental effect of NaN_3_.

## 4. Conclusions

In this work, hybrid materials based on biochar and CeO_2_ were prepared with a simple wet impregnation method, characterized by various techniques and eventually tested for their catalytic activity to activate persulfate and degrade a model antibiotic compound. The main conclusions are as follows:

1. Changing the calcination temperature in the range of –500 °C affected the biochar content and the physicochemical properties of CeO_2_, but more importantly, determines the interactions between biochar and CeO_2_ and, eventually, the catalytic activity.
2. Calcination at 300–350 °C yielded the more active materials for persulfate activation and sulfamethoxazole degradation; the latter following pseudo-first order kinetics with the rate depending on the operating conditions.
3. The water matrix is crucial for process performance since various inorganic and/or organic species can interfere with the surface and/or the target contaminant for the oxidants and the active catalytic sites. Hybrid materials may minimize such competitive interactions that do not exist in model experiments performed in pure water. Should this be the case, hybrid materials are likely to outperform bare biochar in environmentally relevant systems.
4. Radicals and singlet oxygen seem to be the main oxidative species, as indirectly evidenced by means of scavenging experiments.

## Figures and Tables

**Table 1:** Physicochemical characteristics of the prepared samples.

Sample	T Calc (°C)	SSA (m^2^ g^−1^)	pzc	D (XRD) (nm)	% CeO_2_ Content	Eg (eV)	%O_2_ Uptake in TGA
BC/Ce-300	300	119	6.8	29.2	16	3.09	0
BC/Ce-300-5 h	300	110	6.7	20.7	14	3.10	0
BC/Ce-350	350	126	6.5	29.8	22	3.07	1.7
BC/Ce-400	400	69	3.0	18.9	7	3.10	0.5
BC/Ce-500	500	14	3.0	16.0	2	3.12	2.0
